# The identification of two regulatory ESCC susceptibility genetic variants in the *TERT-CLPTM1L* loci

**DOI:** 10.18632/oncotarget.6747

**Published:** 2015-12-24

**Authors:** Liqing Zhou, Guobin Fu, Jinyu Wei, Juan Shi, Wenting Pan, Yanli Ren, Xiangyu Xiong, Jianhong Xia, Yue Shen, Hongliang Li, Ming Yang

**Affiliations:** ^1^ State Key Laboratory of Chemical Resource Engineering, Beijing Laboratory of Biomedical Materials, College of Life Science and Technology, Beijing University of Chemical Technology, Beijing, China; ^2^ Department of Radiation Oncology, Huaian No. 2 Hospital, Huaian, Jiangsu Province, China; ^3^ Department of Oncology, Provincial Hospital Affiliated to Shandong University, Jinan, Shandong Province, China

**Keywords:** TERT, CLPTM1L, polymorphism, esophageal squamous cell carcinoma, susceptibility

## Abstract

The chromosome 5p15.33 *TERT-CLPTM1L* region has been identified by genome-wide association studies as a susceptibility locus of multiple malignancies. However, the involvement of this locus in esophageal squamous cell carcinoma (ESCC) development is still largely unclear. We fine-mapped the *TERT-CLPTM1L* region through genotyping 15 haplotype-tagging single nucleotide polymorphisms (htSNPs) using a two stage case-control strategy. After analyzing 2098 ESCC patients and frequency-matched 2150 unaffected controls, we found that rs2853691, rs2736100 and rs451360 genetic polymorphisms are significantly associated with ESCC risk in Chinese (all *P*<0.05). Reporter gene assays indicated that the ESCC susceptibility SNP rs2736100 locating in a potential *TERT* intronic promoter has a genotype-specific effect on *TERT* expression. Similarly, the *CLPTM1L* rs451360 SNP also showed allelic impacts on gene expression. After measuring *TERT* and *CLPTM1L* expression in sixty-six pairs of esophageal cancer and normal tissues, we observed that the rs2736100 G risk allele carriers showed elevated oncogene *TERT* expression. Also, subjects with the rs451360 protective T allele had much lower oncogene *CLPTM1L* expression than those with G allele in tissue specimens. Results of these analyses underline the complexity of genetic regulation of telomere biology and further support the important role of telomerase in carcinogenesis. Our data also support the involvement of *CLPTM1L* in ESCC susceptibility.

## INTRODUCTION

The chromosome 5p15.33 *TERT-CLPTM1L* region has been repeatedly proved to be a susceptibility locus of multiple malignancies according to genome-wide association studies (GWAS). Independent susceptibility single nucleotide polymorphisms (SNPs) in this region were identified in lung cancer [1–5], melanoma [6], nonmelanoma skin cancer [7,8], glioma [9], bladder cancer [10], pancreatic cancer [11], testicular germ cell cancer [12], estrogen-negative breast cancer [13], ovarian cancer [14] and prostate cancer [15], suggesting that the region harbors several essential elements associating etiology of multiple cancers. The chromosome 5p15.33 region harbors two plausible candidate coding genes *TERT* and *CLPTM1L*. *TERT* encodes the catalytic subunit of telomerase reverse transcriptase [16]. Activated *TERT* transcription in many cancers leads to increased telomerase activity to counteract telomere shortening and promotes malignant transformation of normal cells [17]. *CLPTM1L* encodes the cleft lip and palate-associated transmembrane 1 like protein (also known as cisplatin resistance related protein, *CRR9*). Accumulated evidences demonstrated that CLPTM1L may act as an oncogene in lung and pancreatic cancers [20–22].

As one of the most common and fatal malignant tumors in the world, esophageal squamous cell carcinoma (ESCC) was diagnosed at a relatively high frequency in China [24]. It has been shown that heavy alcohol drinking, tobacco smoking, micronutrient deficiency and dietary carcinogen exposure are risk factors of this lethal disease [25,26]. However, only a portion of exposed individuals develop ESCC, indicating that genetic factors may also impact esophageal malignant transformation. Considering the involvement of the 5p15.33 *TERT-CLPTM1L* locus in ESCC is still largely unclear, we examined the associations between 15 haplotype-tagging SNPs (htSNP) across the *TERT-CLPTM1L* locus and ESCC risk in three large independent hospital-based case-control studies. To investigate the biological function of three ESCC susceptibility SNPs, we examined impacts of these genotypes on *TERT* or *CLPTM1L* expression using luciferase reporter gene assays and inspected the association between these polymorphisms and gene expression in esophageal tissues.

## RESULTS

### Associations between the TERT-CLPTM1L htSNPs and ESCC risk in the discovery case-control set

Genotype distributions of fifteen *TERT-CLPTM1L* genetic variants in the Jiangsu discovery set are showed in Table 1. All observed genotype frequencies in either controls or cases conform to Hardy-Weinberg equilibrium (all *P* > 0.05). Distributions of the all genotypes were then compared among patients and controls. Frequencies of rs2853691, rs2736100 or rs45136 genotypes among patients differed significantly from those among controls (all *P* < 0.05). Logistic regression analyses revealed that rs2853691, rs2736100 and rs451360 SNPs were significantly associated with ESCC risk (rs2853691: allelic OR = 1.50, 95% CI = 1.25-1.80, *P* = 7.0 × 10^−6^; rs2736100: allelic OR = 1.39, 95% CI = 1.17-1.64, *P* = 7.8 × 10^−5^; rs45136: allelic OR = 0.69, 95% CI = 0.52-0.91, *P* = 0.007) (Table 1). However, no statistically significant differences of other htSNPs were observed between cases and controls (all *P* > 0.05) (Table 1). Therefore, no additional genotyping and analyses on these twelve htSNPs were done in the next studies.

**Table 1:** Associations between candidate SNPs in the *TERT-CLPTM1L* locus and risk of ESCC in Jiangsu case-control set

No.	rs ID	Position	Base change	MAF^1^	Genotype (588 cases and 600 controls)				*P*^3^
					Common^2^	Heterozygous^2^	Rare^2^	OR(95%CI)^3^	
**1**	**rs2853691**	**1305950**	**T>C**	**0.257**	**44.2/54.6**	**43.5/39.4**	**12.3/6.0**	**1.50 (1.25-1.80)**	**7.0 × 10^−6^**
2	rs2736122	1310621	G>A	0.058	87.8/89.1	12.1/10.3	0.1/0.6	1.07 (0.75-1.52)	0.701
3	rs2075786	1319310	A>G	0.128	74.5/76.3	24.0/21.8	1.5/1.9	1.06 (0.83-1.36)	0.621
4	rs4246742	1320356	T>A	0.355	43.8/40.9	44.6/47.0	11.7/12.0	0.93 (0.78-1.10)	0.397
5	rs4975605	1328528	C>A	0.053	90.5/89.7	9.3/10.1	0.1/0.2	0.90 (0.61-1.33)	0.586
**6**	**rs2736100**	**1339516**	**A>C**	**0.405**	**28.1/35.7**	**46.7/47.6**	**25.2/16.7**	**1.39 (1.17-1.64)**	**7.8 × 10^−5^**
7	rs2853676	1341547	C>T	0.126	75.3/75.9	23.0/22.9	1.7/1.1	1.05 (0.82-1.35)	0.664
8	rs2736098	1347086	C>T	0.392	40.8/41.0	39.7/39.6	19.5/19.4	1.01 (0.85-1.19)	0.919
9	rs2853668	1353025	G>T	0.293	49.5/49.8	42.1/42.0	8.4/8.3	0.99 (0.83-1.19)	0.927
10	rs2735845	1353584	C>G	0.328	45.2/45.3	44.5/43.7	10.3/11.0	0.99 (0.83-1.18)	0.890
11	rs6554759	1370102	A>G	0.045	90.3/91.1	9.4/8.7	0.3/0.2	1.12 (0.76-1.66)	0.554
**12**	**rs451360**	**1372680**	**C>A**	**0.117**	**83.9/78.2**	**15.3/20.2**	**0.8/1.6**	**0.69 (0.52-0.91)**	**0.007**
13	rs380286	1373247	G>A	0.125	77.4/76.5	21.5/22.0	1.1/1.5	0.94 (0.73-1.21)	0.612
14	rs402710	1373722	C>T	0.325	44.5/45.8	44.9/43.4	10.6/10.8	1.03 (0.86-1.22)	0.764
15	rs452932	1383253	T>C	0.160	72.6/70.9	25.6/26.1	1.8/2.9	0.90 (0.71-1.13)	0.352

Note: ESCC, esophageal squamous cell carcinoma; MAF, minor allele frequency; OR, odds ratios; 95%CI, 95% confident intervals.

1 MAF in healthy controls.

2 % of case/% of control.

3 Allelic OR calculated by logistic regression.

### TERT-CLPTM1L rs2853691, rs2736100 and rs45136 polymorphisms contribute to ESCC susceptibility

Associations between genotypes of rs2853691, rs2736100 and rs45136 genetic variants and ESCC risk were calculated using unconditional logistic regression analyses in Jiangsu set (Table 2). The rs2853691 G allele was showed to be risk allele; subjects having the AG or GG genotype had an OR of 1.19(95% CI = 0.88-1.60, *P* = 0.214) or 1.40(95% CI = 1.10-1.77, *P* = 0.006) for developing ESCC, respectively, compared with subjects having the AA genotype. It was observed that the odds of having the rs2736100 TG or GG genotype in patients was 1.32 (95% CI = 0.98-1.76, *P* = 0.066) or 1.37 (95% CI = 1.14-1.64, *P* = 7.3 × 10^−4^) compared with the TT genotype. Moreover, a significantly decreased OR was associated with the rs451360 GT genotype (OR = 0.67, 95% CI = 0.48-0.93, *P* = 0.018) but not the TT genotype (OR = 0.72, 95% CI = 0.41-1.29, *P* = 0.275). All ORs were adjusted for sex, age, smoking and alcohol drinking status.

**Table 2:** Genotype frequencies of rs2853691 A>G, rs2736100 T>G and rs451360 G>T SNPs in the *TERT-CLPTM1L* locus among cases and controls and their association with ESCC risk

Studies	rs2853691 A>G					rs2736100 T>G				
	Genotypes	Cases No. (%)	Controls No. (%)	OR^1^(95% CI)	*P*^1^	Genotypes	Cases No. (%)	Controls No. (%)	OR^1^(95% CI)	*P*^1^
		*n* = 588	*n* = 600				*n* = 588	*n* = 600		
Jiangsu set	AA	260(44.2)	328(54.6)	1.00		TT	165(28.1)	214(35.7)	1.00	
	AG	256(43.5)	236(39.4)	1.19(0.88-1.60)	0.214	TG	275(46.7)	285(47.6)	1.32(0.98-1.76)	0.066
	GG	72(12.3)	36(6.0)	1.40(1.10-1.77)	0.006	GG	148(25.2)	101(16.7)	1.37(1.14-1.64)	7.3 × 10^−4^
		*n* = 1000	*n* = 1000				*n* = 1000	*n* = 1000		
Shandong set	AA	503(50.3)	557(55.7)	1.00		TT	279(27.9)	363(36.3)	1.00	
	AG	418(41.8)	393(39.3)	1.17(0.96-1.43)	0.116	TG	472(47.2)	475(47.5)	1.24(0.99-1.54)	0.051
	GG	79(7.9)	50(5.0)	1.30(1.07-1.59)	0.010	GG	249(24.9)	162(16.2)	1.48(1.29-1.70)	2.2 × 10^−6^
		*n* = 510	*n* = 550				*n* = 508	*n* = 547		
Hebei set	AA	270(52.9)	315(57.3)	1.00		TT	163(32.1)	222(40.6)	1.00	
	AG	194(38.0)	202(36.7)	1.08(0.83-1.41)	0.584	TG	244(48.0)	241(44.0)	1.45(1.09-1.92)	0.011
	GG	46(9.1)	33(6.0)	1.29(1.01-1.64)	0.048	GG	101(19.9)	84(15.4)	1.32(1.10-1.59)	3.2 × 10^−4^
		*n* = 2098	*n* = 2150				*n* = 2096	*n* = 2147		
Pooled	AA	1033(49.2)	1200(55.8)	1.00		TT	607(29.0)	799(37.2)	1.00	
	AG	868(41.4)	831(38.7)	1.16(1.01-1.32)	0.036	TG	991(47.2)	1001(46.6)	1.32(1.44-1.53)	2.1 × 10^−4^
	GG	197(9.4)	119(5.5)	1.32(1.16-1.50)	2.2 × 10^−5^	GG	498(23.8)	347(16.2)	1.41(1.29-1.55)	4.0 × 10^−8^

Studies	rs451360 G>T				
	Genotypes	Cases No. (%)	Controls No. (%)	OR^1^(95% CI)	*P*^1^
		*n* = 588	*n* = 600		
Jiangsu set	GG	493(83.9)	469(78.2)	1.00	
	GT	90(15.3)	121(20.2)	0.67(0.48-0.93)	0.018
	TT	5(0.8)	10(1.6)	0.72(0.41-1.29)	0.275
		*n* = 1000	*n* = 1000		
Shandong set	GG	819(81.9)	760(76.0)	1.00	
	GT	176(17.6)	222(22.2)	0.70(0.55-0.89)	0.003
	TT	5(0.5)	18(1.8)	0.56(0.33-0.95)	0.033
		*n* = 510	*n* = 550		
Hebei set	GG	416(81.6)	417(75.8)	1.00	
	GT	90(17.6)	124(22.6)	0.68(0.49-0.93)	0.017
	TT	4(0.8)	9(1.6)	0.59(032-1.08)	0.086
		*n* = 2098	*n* = 2150		
Pooled	GG	1728(82.3)	1646(76.6)	1.00	
	GT	356(17.0)	467(21.7)	0.68(0.58-0.81)	6.3 × 10^−6^
	TT	14(0.7)	37(1.7)	0.61(0.44-0.85)	0.003

Note: ESCC, esophageal squamous cell carcinoma; OR, odds ratio; CI, confidence interval.

1 Data were calculated by logistic regression with adjustment for age, sex, smoking and drinking status.

In two validation sets (Shandong set and Hebei set), the significant associations between rs2853691, rs2736100 and rs45136 SNPs and ESCC risk were also observed (Table 2). Individuals with rs2853691 GG genotype showed significantly increased ESCC risk compared with those with rs2853691 AA genotype in both validation sets (OR_Shandong_ = 1.30, 95% CI = 1.07-1.59, *P* = 0.010; OR_Hebei_ = 1.29, 95% CI = 1.01-1.64, *P* = 0.048). Carriers of rs2736100 GG genotype showed significantly elevated risks developing ESCC compared with rs2736100 TT carriers (OR_Beijing_ = 1.48, 95% CI = 1.29-1.70, *P* = 2.2 × 10^−6^; OR_Hebei_ = 1.32, 95% CI = 1.10-1.59, *P* = 3.2 × 10^−4^). The odds of having the rs451360 GT or TT genotype in patients was 0.70 (95% CI = 0.55-0.89, *P*=0.003) or 0.56 (95% CI = 0.33-0.95, *P* = 0.033) compared with the AA genotype in Shandong set. However, only rs451360 GT genotype was significantly associated with ESCC risk (OR = 0.68, 95% CI = 0.49-0.93, *P* = 0.017) in Hebei validation set.

In the pooled analyses, we found that the rs2853691 AG or GG genotype carriers had a 1.16-fold or 1.32-fold increased risk to develop ESCC compared to the AA genotype carriers (95% CI = 1.01-1.32, *P* = 0.036 or 95% CI = 1.16-1.50, *P* = 2.2 × 10^−5^) (Table 2). Intriguingly, either rs2736100 TG or GG genotype was significantly associated with ESCC risk (OR = 1.32, 95% CI = 1.44-1.53, *P* = 2.1 × 10^−4^; OR = 1.41, 95% CI = 1.29-1.55, *P* = 4.0 × 10^−8^) compared to the TT genotype. Similarly, subjects having the rs451360 GT or TT genotype had an OR of 0.68 (95%CI = 0.58-0.81, *P* = 6.3 × 10^−6^) or 0.61 (95%CI = 0.44-0.85, *P* = 0.003) for developing ESCC compared with individual having the GG genotype.

### Stratified analyses of associations between rs2853691, rs2736100 or rs451360 SNP and ESCC risk

The risk of ESCC associated with the rs2853691, rs2736100 or rs451360 SNP was further investigated by stratifying for age, sex, smoking and alcohol drinking status using the combined data of three case-control sets (Table 3). For rs2853691, a significantly increased risk of ESCC associated with the rs2853691 GG genotype compared with the AA genotype was observed for both groups stratified by sex, smoking and drinking status (all *P* < 0.05) or the group aged 58 years or younger (*P* = 5.4 × 10^−5^). Additionally, the rs2853691 AG genotype was only associated with ESCC risk in the male group (*P* = 0.026), the smoking group (*P* = 0.008) or the drinking group (*P* = 0.013). For rs2736100, significant associations between TG or GG genotype and ESCC risk were observed in all stratified groups (all *P* < 0.05), but not in the drinking group (*P* = 0.974). There was a significantly multiplicative gene-drinking interaction (*P*_interaction_ = 0.012). For rs451360, the TT genotype was only associated with ESCC risk in the male group (*P* = 0.006), the group aged 58 years or younger (*P* = 0.016), the non-smoking group (*P* = 0.025) or the drinking group (*P* = 0.005). However, significant associations between the rs451360 GT genotype and ESCC risk were observed in all stratified groups (all *P* < 0.05), but not in the female group (*P* = 0.326).

**Table 3:** Risk of ESCC associated with rs2853691 A>G, rs2736100 T>G and rs451360 G>T genotypes by age, sex, smoking, and drinking status

Variable	rs2853691 A>G							
	AA^1^	AG^1^	OR^2^ (95% CI)	*P*	GG^1^	OR^2^ (95% CI)	*P*	*P*_interaction_^3^
Sex								0.842
Male	789/918	661/624	1.19(1.02-1.38)	0.026	137/93	1.27(1.10-1.47)	0.001	
Female	244/288	207/207	0.88(0.62-1.25)	0.475	60/26	1.41(1.05-1.91)	0.025	
Age (year)								0.363
≤58	535/606	431/418	1.08(0.89-1.32)	0.431	109/54	1.48(1.22-1.79)	5.4 × 10−5	
>58	498/594	437/413	1.21(0.99-1.46)	0.055	88/65	1.19(0.99-1.43)	0.055	
Smoking								0.096
No	290/721	196/499	0.98(0.79-1.22)	0.863	42/68	1.27(1.03-1.56)	0.027	
Yes	743/479	672/332	1.27(1.07-1.52)	0.008	155/51	1.36(1.14-1.61)	0.001	
Drinking								0.980
No	465/709	373/507	1.06(0.87-1.29)	0.582	85/63	1.44(1.19-1.74)	1.6 × 10−4	
Yes	568/491	495/324	1.27(1.05-1.54)	0.013	112/56	1.24(1.04-1.48)	0.017	

Variable	rs2736100 T>G							*P*_interaction_^3^
	TT^1^	TG^1^	OR^2^ (95% CI)	*P*	GG^1^	OR^2^ (95% CI)	*P*	
Sex								0.253
Male	478/610	750/762	1.25(1.06-1.47)	0.008	357/262	1.92(1.28-2.88)	0.002	
Female	129/189	241/239	1.92(1.28-2.88)	0.002	141/85	1.77(1.40-2.24)	1.9 × 10^−6^	
Age (year)								0.606
≤58	311/411	502/497	1.27(1.02-1.57)	0.030	262/168	1.46(1.28-1.68)	6.1 × 10^−8^	
>58	296/388	489/504	1.36(1.10-1.68)	0.005	236/179	1.39(1.22-1.59)	1.2 × 10^−6^	
Smoking status								0.065
No	138/479	244/600	1.48(1.15-1.89)	0.002	145/206	1.57(1.36-1.82)	1.4 × 10^−9^	
Yes	469/320	747/401	1.27(1.05-1.54)	0.015	353/141	1.28(1.13-1.45)	8.3 × 10^−5^	
Drinking status								0.012
No	221/486	463/588	1.90(1.52-2.37)	1.9 × 10^−6^	238/203	1.62 (1.41-1.85)	2.6 × 10^−8^	
Yes	386/313	528/413	1.00(0.81-1.22)	0.974	260/144	1.22(1.07-1.39)	0.003	

Variable	rs451360 G>T							*P*_interaction_^3^
	GG^1^	GT^1^	OR^2^ (95% CI)	*P*	TT^1^	OR^2^ (95% CI)	*P*	
Sex								0.085
Male	1305/1230	271/375	0.65(0.54-0.78)	3.6 × 10^−6^	11/30	0.60(0.42-0.86)	0.006	
Female	423/416	85/92	0.80(0.52-1.24)	0.326	3/7	0.63(0.26-1.54)	0.310	
Age (year)								0.895
≤58	881/822	187/233	0.71(0.56-0.90)	0.005	7/23	0.57(0.36-0.90)	0.016	
>58	847/824	169/234	0.63(0.50-0.80)	1.3 × 10^−4^	7/14	0.66(0.41-1.06)	0.087	
Smoking								0.932
No	443/1001	82/263	0.69(0.52-0.90)	0.007	3/24	0.50(0.27-0.91)	0.025	
Yes	1285/645	274/204	0.69(0.56-0.85)	4.4 × 10^−4^	11/13	0.67(0.44-1.01)	0.057	
Drinking								0.374
No	777/1004	139/258	0.70(0.55-0.90)	0.006	7/17	0.74(0.46-1.20)	0.224	
Yes	951/642	217/209	0.69(0.55-0.86)	0.001	7/20	0.53(0.34-0.82)	0.005	

Note: ESCC, esophageal squamous cell carcinoma; OR, odds ratio; CI, confidence interval.

1 Number of case patients with genotype/number of control subjects with genotype.

2 Data were calculated by logistic regression, adjusted for sex, age, smoking, and drinking status, where it was appropriate.

3 *P* values for gene-environment interaction were calculated using the multiplicative interaction term in SPSS software.

### Functional relevance of TERT rs2736100 and CLPTM1L rs451360 genetic variants on gene expression

**Figure 1:**
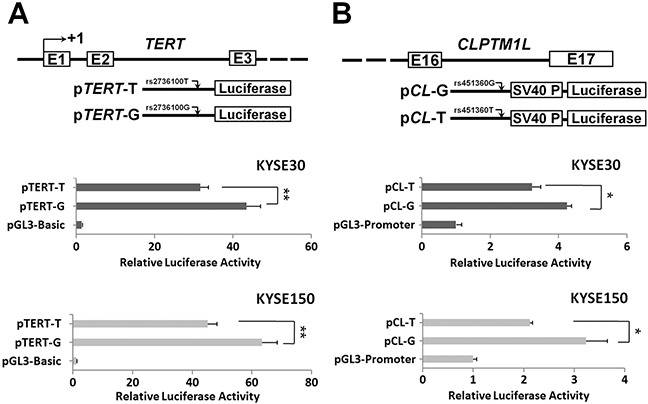
Transient luciferase reporter gene expression assays with constructs containing different rs2736100 allele of the TERT intron 2 region (A) or different rs451360 allele of the CLPTM1L intron 16 region (B) in KYSE30 cells or KYSE150 cells pRL-SV40 were cotransfected with these contructs to standardize transfection efficiency. Fold-changes were detected by defining the luciferase activity of cells co-transfected with pGL3-basic as 1. All experiments were performed in triplicates in three independent transfection experiments and each value represents mean ± SD. Compared with pGL3-Basic transfected cells, *P<0.05; **P<0.01.

Considering the chromosome location of the three ESCC susceptibility SNPs, we only investigated the impacts of *TERT* rs2736100 and *CLPTM1L* rs451360 SNPs on gene expression. The rs2736100 variant locates in the intron 2 region of *TERT*. As shown in Figure 1A, reporter gene assays demonstrated that the intron 2 segment containing the rs2736100 flanking sequence showed promoter activities in KYSE30 and KYSE150 ESCC cells. Moreover, the *TERT* rs2736100G allelic reporter construct (pTERT-G) showed significantly higher luciferase activities compared to the rs920778T allelic reporter construct (pTERT-T) (both *P*<0.01) (Figure 1A). We next examined whether the ESCC susceptibility SNP rs451360 has an allele-specific effect on the intronic enhancer activity on *CLPTM1L* expression in ESCC. Either KYSE30 cells or KYSE150 cells transfected with the *CLPTM1L* pCL-T allelic plasmid showed significantly lower luciferase activities compared to cells expressing pCL-G allelic reporter construct (both *P*<0.05) (Figure 1B).

**Figure 2:**
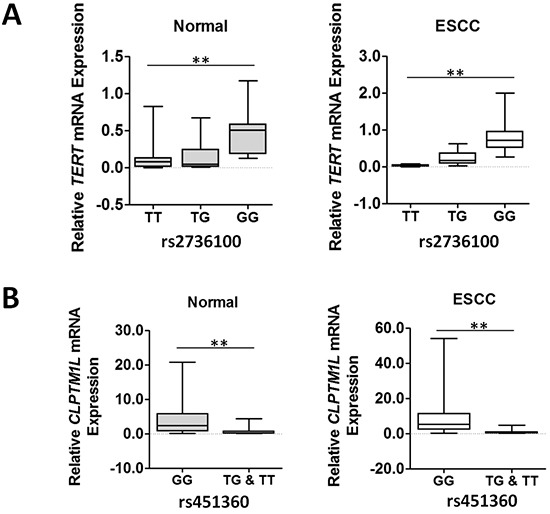
TERT or CLPTM1L mRNA expression in normal and cancerous esophageal tissues grouped by rs2736100 or rs451360 genotypes The expression of individual *TERT* or *CLPTM1L* mRNA was calculated relative to expression of *β-actin* using the 2^−dCt^ method. **P<0.01.

We next examined whether these two ESCC susceptibility SNPs has an allele-specific effect on gene expression in esophagus tissues. As shown in Figure 2A, we found that subjects with the rs920778 TT genotype had significantly lower *TERT* mRNA levels (mean ± SE) than those with the GG genotypes in normal esophagus tissues (0.128 ± 0.047 [*n*=18] vs. 0.493 ± 0.078 [*n*=17], *P*<0.01) or ESCC tissues (0.030 ± 0.006 [*n*=18] vs. 0.847 ± 0.120 [*n*=17], *P*<0.01). Similar results were observed when the *CLPTM1L* mRNA levels were compared between rs451360 GT+TT and GG genotypes in both normal tissues (0.713 ± 0.266 [*n*=17] vs. 4.810 ± 0.810 [*n*=49], *P*<0.01) and ESCC specimens (1.059 ± 0.346 [*n*=17] vs. 10.650 ± 1.922 [*n*=49], *P*<0.01).

## DISCUSSION

In the current study, we systematically examined the impacts of SNPs in the *TERT-CLPTM1L* loci on ESCC susceptibility via a case-control design as well as gene expression of *TERT* or *CLPTM1L in vitro* and *in vivo*. After genotyping 15 htSNPs in the discovery stage, we identified three ESCC susceptibility genetic polymorphisms (rs2853691, rs2736100 and rs451360) which were validated in two validation case-control sets. Reporter gene assays indicated that the ESCC susceptibility SNP rs2736100 locating in a potential *TERT* intronic promoter has a genotype-specific effect on *TERT* expression. Similarly, the *CLPTM1L* rs451360 polymorphism also showed allelic effects on gene expression. Genotype-phenotype correlation data supported the regulatory role of these two genetic variants in *TERT* or *CLPTM1L* gene expression *in vivo*. Our observations support the hypothesis that genetic polymorphisms in oncogene regulatory elements might explain a part of ESCC genetic basis besides those genetic variants identified by GWAS [27–31].

Interestingly, *TERT* rs2736100 has been found to be associated with risk of lung cancer [1,2,24], glioma[9], testicular cancer [12],colorectal cancer [33], acute myeloid leukemia [34], pancreatic cancer [35] and bladder cancer [36]. However, its involvement in ESCC etiology is still largely unclear. To the best of our knowledge, this is the first case-control study to investigate the association between the *TERT* rs2736100 polymorphism and ESCC risk. We observed a significantly increased ESCC risk among individuals with *TERT* rs2736100 G allele compared to carriers of rs2736100 T allele. It has been reported that increased TERT expression in ESCC tissues were observed compared to normal tissues [18,19], which indicated the oncogene nature of *TERT* in ESCC. Since rs2736100 G allele is associated with elevated *TERT* expression, the associations between the polymorphism and increased cancer risk are biologically plausible.

*CLPTM1L* appears to act as an oncogene with significantly increased expression in malignant tissues [20–23]. In line with this, CLPTM1L silencing by miR-494 can inhibit cell growth and invasion and induce ESCC cell apoptosis [23]. The *CLPTM1L* rs451360 polymorphism has been associated with decreased risk of lung cancer among different ethnic populations, with T allele as a protective allele [5,37-41]. Here, we provided first evidences that rs451360 SNP also play a part in ESCC susceptibility, which are unlikely to be attributable to unknown confounding factors due to having relatively large sample sizes, significantly increased odd ratios with small *P* values. Additionally, our genotype-phenotype correlation data between the rs451360 genetic variant and gene expression supports the case-control study since the protective T allele carriers showing less oncogene *CLPTM1L* expression. Since the TT genotype of the functional rs451360 SNP is relatively rare (about 1-2% among common populations), the potential clinical translation of this genetic variant might be compromised.

In conclusion, we demonstrated that there are three genetic polymorphisms (rs2853691, rs2736100 and rs451360) in the *TERT-CLPTM1L* loci are significantly associated with ESCC risk in Chinese populations. Our results underline the complexity of genetic regulation of telomere biology and further support the important role of telomerase in carcinogenesis. Our data also support the involvement of *CLPTM1L* in ESCC susceptibility. These results may lead to better understanding of ESCC etiology in different populations.

## MATERIALS AND METHODS

### Study subjects

This study consisted of three case-control sets: (a) Jiangsu set: 588 ESCC cases from Huaian No. 2 Hospital (Huaian, Jiangsu Province, China) and sex- and age-matched 600 controls. (b) Shandong set: 1000 cases with ESCC from Shandong Cancer Hospital, Shandong Academy of Medical Sciences (Jinan, Shandong Province, China) and sex- and age-matched (± 5 years) 1000 healthy controls. (c) Hebei study: 510 ESCC patients from Bethune International Peace Hospital (Shijiazhuang, Hebei Province, China) and 550 sex- and age-matched healthy controls. Sixty-six pairs of ESCC specimens and esophagus normal tissues adjacent to the tumors were obtained from surgically removed specimens of patients in Bethune International Peace Hospital and Huaian No. 2 Hospital. All individuals were ethnic Han Chinese. At recruitment, the informed consent was obtained from each subject. The detailed information on subject recruitments can be found in Supplementary Table S1 and our previous studies [42–44]. This study was approved by the institutional Review Boards.

### SNP selection and genotyping

The *TERT-CLPTM1L* gene loci cover a 91716bp region of chromosome 5p15.33 and contain a great number of SNPs. An htSNP approach was utilized to analyze the *TERT-CLPTM1L* genetic polymorphisms globally [45]. Genotyped HapMap SNPs among Han Chinese and Japanese populations (HapMap Rel 21, NCBI B36) with a minor allele frequency >5% were included in the selection. The htSNPs were chosen in a 95716bp region (91716bp *TERT-CLPTM1L* loci and 2kb up-stream as well as 2kb down-stream regions of the *TERT-CLPTM1L* gene loci). Using a method described previously with the sample size inflation factor, Rh^2^, of ≥ 0.8, fifteen htSNPs were selected with Haploview 4.2 software on a block-by-block basis (Supplementary Table S2).

*TERT-CLPTM1L* htSNPs were genotyped through the MassArray system (Sequenom Inc., San Diego, California, USA). A 5% blind, random DNA samples was analyzed in duplicates and the reproducibility was 99%. To reduce the costs of the study, we genotyped the *TERT-CLPTM1L* rs2853691 A>G, rs2736100 T>G and rs451360 G>T SNPs in two validation sets using the PCR-based restriction fragment length polymorphism (RFLP) as described in Supplementary Table S3. A 5% samples were genotyped by two investigators and the reproducibility was 98.0%.

### Luciferase reporter gene constructs

Specific primer pairs (Supplementary Table S4) with the *Kpn*I and *Xho*I restriction sites were used to amplify the intron 2 segment of *TERT* (chr.5: 1319429∼1319865 bp [GRCh38.p2] including the rs2736100 flanking region) from human genomic DNA carrying *TERT* rs2736100 TT genotype or GG genotype. Similarly, the intron 16 segment of *CLPTM1L* (chr.5: 1285954∼1286844 bp [GRCh38.p2] including the rs451360 flanking region) was amplified with human genomic DNA carrying *CLPTM1L* rs451360 GG genotype or TT genotype. The PCR products were then digested with *Kpn*I and *Xho*I (New England Biolabs) and ligated into an appropriately digested pGL3-Basic vector (*TERT*) or pGL3-Promoter vector (*CLPTM1L*). The resultant *TERT* reporter gene plasmids were designated pTERT-T or pTERT-G, which were only different at rs2736100 polymorphic site. The resultant *CLPTM1L* reporter gene plasmids were named as pCL-T or pCL-G, which were identical except for the different allele at rs451360 polymorphic site. Restriction analysis and complete DNA sequencing confirmed the orientation and integrity of these constructs.

### Dual luciferase reporter assays

KYSE30 and KYSE150 ESCC cells were transfected with both reporter constructs (pGL3-Basic, pTERT-T, pTERT-G, pGL3-Promoter, pCL-T or pCL-G) and pRL-SV40 (Luciferase Assay System; Promega). Dual luciferase activities were determined at 48h after transfection as previously described [46,47]. For each plasmid construct, three independent transfection experiments were performed, and each was done in triplicates.

### Real-time analysis of TERT and CLPTM1L mRNA

Total cellular RNA was isolated from sixty-six pairs of ESCC specimens and esophagus normal tissues adjacent to the tumors with TRIzol Reagent (Invitrogen) and converted to cDNA using the PrimeScript RT Master Mix (TAKARA). *TERT* and *CLPTM1L* mRNA expression in cancerous and normal esophagus tissues was examined using the TaqMan real-time quantity PCR method. Relative gene expression quantization for *TERT* (ABI, Assay ID Hs00972656_m1) and *CLPTM1L* (ABI, Assay ID Hs00363947_m1) was calculated using *β-actin* (ABI, Assay ID 4333762T) as an internal reference gene was carried out using the ABI 7500 real-time PCR system in triplicates.

### Statistics

Pearson's χ^2^ test was used to examine the differences in demographic variables, smoking status, drinking status, and genotype distributions of rs2853691, rs2736100 or rs451360 SNP between ESCC cases and healthy controls. The associations between rs2853691, rs2736100 or rs451360 genotypes and ESCC risk were estimated by odds ratios and their 95% confidence intervals computed by logistic regression models. All ORs were adjusted for age, sex, smoking or drinking status, where it was appropriate. A *P* value of less than 0.05 was used as the criterion of statistical significance, and all statistical tests were two-sided. All analyses were performed using SPSS 16.0 (SPSS Inc.).

## SUPPLEMENTARY TABLES


