# Supplementary material for: The identification of two regulatory ESCC susceptibility genetic variants in the TERT-CLPTM1L loci
Source: Oncotarget. 2015 Dec 24;7(5):5495–506. doi: 10.18632/oncotarget.6747 (PMC4868701; doi:10.18632/oncotarget.6747)
Supplement: Supplementary file 1 [file oncotarget-07-5495-s001.pdf]

SUPPLEMENTARY TABLES

Supplementary Table S1: Distribution of selected characteristics among ESCC cases and controls

Variable	Jiangsu case-control set(discovery set)			Shandong case-control set(validation set 1)			Hebei case-control set(validation set 2)		
	Cases	Controls	*P*-value [a]	Cases	Controls	*P*-value[a]	Cases	Controls	*P*-value[a]
	No. (%)	No. (%)		No. (%)	No. (%)		No. (%)	No. (%)	
	588	600		1000	1000		510	550	
Sex			0.678			0.426			0.218
Male	413(70.2)	428(71.3)		776(77.6)	761(76.1)		398(78.0)	446(81.1)	
Female	175(29.8)	172(28.7)		224(22.4)	239(23.9)		112(22.0)	104(18.9)	
Age (year)[2]			0.725			0.474			0.433
≤ 59(or 56)	288(49.0)	300(50.0)		516(51.6)	500(50.0)		271(53.1)	279(50.7)	
> 59(or 56)	300(51.0)	300(50.0)		484(48.4)	500(50.0)		239(46.9)	271(49.3)	
Smoking status			< 0.001			< 0.001			< 0.001
No	151(25.7)	397(66.2)		248(24.8)	604(60.4)		129(25.3)	287(52.2)	
Yes	437(74.3)	203(33.8)		752(75.2)	396(39.6)		381(74.7)	263(47.8)	
Drinking status			< 0.001			< 0.001			< 0.001
No	254(43.2)	358 (59.7)		447(44.7)	599(59.9)		222(43.5)	322(58.5)	
Yes	334(56.8)	242(40.3)		553(55.3)	401(40.1)		288(56.5)	228(41.5)	

Note: ESCC, esophageal squamous cell carcinoma.
[1]Two-sided χ^2 test.
[2]Median ages of cases for Shandong case-control set, Jiangsu case-control set and Hebei case-control set are 56, 59 and 59 years.

Supplementary Table S2: HapMap tag-SNPs in *TERT-CLPTM1L* locus with HCB+JPT data of HapMap Rel 21

Test	Alleles captured
rs452932	rs27070,rs4975615,rs421629,rs465498,rs4975616,rs31489,rs452384,rs467095,rs466502,rs31484,rs452932
rs6554759	rs10073340,rs1801075,rs6554759
rs451360	rs4635969,rs451360
rs402710	rs402710,rs401681
rs2736122	rs2736122
rs2075786	rs2075786
rs2736100	rs2736100
rs2853691	rs2853691
rs2736098	rs2736098
rs380286	rs380286
rs2853668	rs2853668
rs2735845	rs2735845
rs4246742	rs4246742
rs4975605	rs4975605
rs2853676	rs2853676

Note: SNP, single nucleotide polymorphism; HCB, Han Chinese Beijing; JPT, Japanese in Tokyo.

Supplementary Table S3: Genotyping *TERT-CLPTM1L* rs2853691 A > G, rs2736100 T > G and rs451360 G > T using PCR-based restriction fragment length polymorphism

SNPs	PCR primers (5′→ 3′)	Restriction endonucleases	Restriction fragmentlength (bp)
rs2853691 (A > G)	F: GGATGATGGAGGGCCTGGCC<br>R: CCAGCCAGGCTTCCCCATCTTC	MspI	AA: 114<br>AG: 114+95+19GG: 95+19
rs2736100 (T > G)	F: GCAGGGCGGGGGCAAAGGTA<br>R: CACCCCCCAGCCTGTGTGCTGT	RsaI	TT: 132<br>TG: 132+111+21<br>GG: 111+21
rs451360 (G > T)	F: TGCCTAGGTCCGGGGTGCCG<br>R: ACCATGCCAGCCTCATGCTGGCT	*Hae*III	TT: 121<br>GT: 121+101+20<br>GG: 101+20

Abbreviations: TERT, Telomerase reverse transcriptase; CLPTM1L, CLPTM1-like; PCR, polymerase chain reaction; SNP, single nucleotide polymorphisms; bp, base pair.
Note: The mismatch base in PCR primers was underlined.

Supplementary Table S4: PCR primers used in construction of luciferase reporter plasmids

Plasmid constructs	PCR clone primers (5′→ 3′)
pTERT	F: CGGGGTACCGTGTAATGGCACAATCTCGG<br>R: CCGCTCGAGCAAGCAGAAGGGAGGAAGC
p-CL	F: CGGGGTACCGCAAGCACTGCCCTCACCTC<br>R: CCGCTCGAGCCAACTGCACCCGAACAAAGA

Abbreviations: PCR, polymerase chain reaction.
